# A nonlocal metasurface for optical edge detection in the far-field

**DOI:** 10.1515/nanoph-2025-0373

**Published:** 2025-09-19

**Authors:** Doyoon Lee, Huu Lam Phan, Minkyung Kim

**Affiliations:** Department of Mechanical and Robotics Engineering, 65419 Gwangju Institute of Science and Technology (GIST), 61005 Gwangju, Republic of Korea; Mechatronics and Robotics Research Group, Faculty of Engineering and Technology, Nguyen Tat Thanh University, Ho Chi Minh 700000, Vietnam; 65419 GIST InnoCORE AI-Nano Convergence Institute for Early Detection of Neurodegenerative Diseases, Gwangju Institute of Science and Technology, Cheomdangwagiro 123, 61005 Gwangju, Republic of Korea

**Keywords:** analog optical imaging, spatial differentiation, negative index, photonic crystal slab, nonlocal optics

## Abstract

Recent studies on nonlocal metasurfaces have shown possibilities of optical image processing, such as edge detection, without the need for Fourier optics and have significantly reduced the form factor. However, the analog edge detection using nonlocal metasurfaces still requires the use of multiple lenses to image the edge-enhanced results, and the edge-detected image generally suffers from image distortion originating from the free space propagation before reaching the detector otherwise. In this work, we propose a nonlocal metasurface that not only enhances the edge features but also delivers them to the far-field with considerably less distortion by combining the conventional edge detection metasurface with a uniaxial slab that has a transfer function of the free space with a negative propagation length. This space expander cancels out the diffraction of the edge-enhanced image that occurs during the free space propagation, the thickness of which reaches several orders of magnitude larger than the slab thickness. This nonlocal metasurface for the far-field edge detection will open a path towards compact optical systems for high-quality analog image processing.

## 1 Introduction

Recent advances in nanotechnology have opened an era of flat optics, in which light can be tailored with extreme degrees of freedom while transmitting through thin planar structures [1]. Whereas the thickness of such structures is comparable to or even less than the wavelength, flat optics has successfully reproduced many optical phenomena and applications that have been otherwise achieved through propagation of bulk media, the thickness of which reaches several tens or hundreds of wavelengths [2], [3], [4]. The center of this reproduction is a metasurface, an array of subwavelength-scale antennas that are designed to selectively control various light properties such as amplitude [5], phase [6], [7], [8], polarization [9], [10], and temporal/spatial frequencies [11], [12]. Designs of such metasurfaces mostly rely on a local approximation that the transmission and reflection are fully determined by the position [13].

Recently, it has been pointed out that nonlocality, i.e., nonlocal manipulation of light, can expand the coverage of flat optics and further compactify optical systems [14], [15]. In this regime, transmitted or reflected light at a given point is not solely determined by the transmission/reflection and incident light at the point [16], [17], [18], but is affected by the angular transmission/reflection profiles of the entire metasurface mediated by, for example, laterally guided waves [19], [20], [21], [22]. As such, metasurfaces with strong nonlocality enable wave vector (**k**) space operations without the need for Fourier optics components [23], [24], [25], [26]. One representative example is analog optical edge detection [27], which features the high **k** components, i.e., the edges, of a given image using the **k**-dependent amplitude transfer function (ATF). Its linear or quadratic relation with respect to **k** provides spatial differentiation (∇) or Laplace (∇^2^) operations, respectively, in a purely analog manner in the speed of light [11], [28], [29]. The ATF that has a gradually decreasing amplitude as **k** decreases and vanishes as **k** → 0 eliminates the zero spatial frequency components and enhances the edges [28], [30], [31], [32]. With local metasurfaces [33], photonic crystals [29], and the spin Hall effect of light (SHEL) [34], [35], nonlocal metasurfaces open a new route toward compact, analog edge detection.

**Figure 1:**
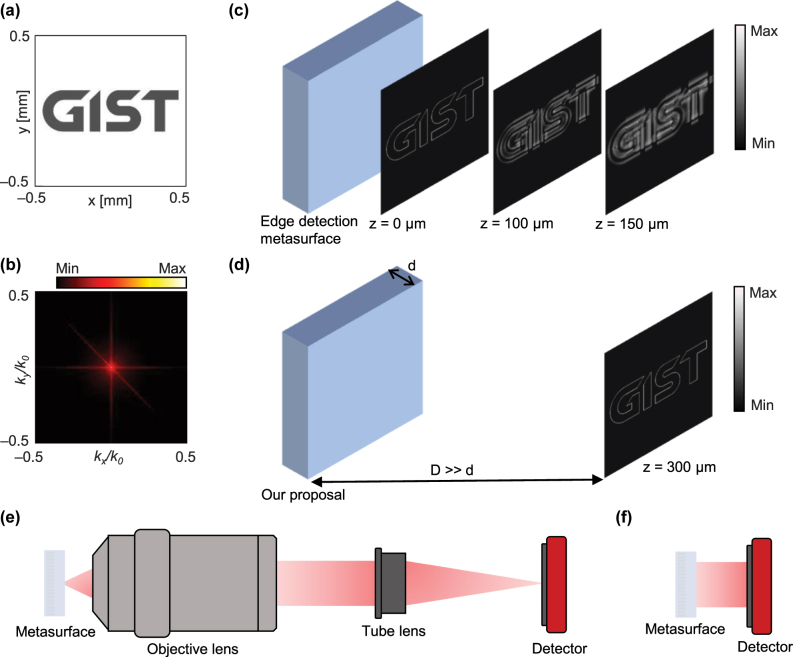
Schematic of edge detection and importance of far-field edge detection. (a) Input image and (b) its **k** space distribution. (c) Evolution of simulated edge-detected images during propagation using the conventional edge detection metasurface and (d) our metasurface designed for the far-field edge detection. Comparison between the optical setups of (e) conventional and (f) our metasurface for edge detection. All intensity distributions are shown on a logarithmic color scale.

One problem with this edge detection using the nonlocal metasurface, however, is that the edge-enhanced image is generally formed right after the metasurface and is rapidly distorted during propagation because of its high **k** nature (Figure 1(a)–(c)). This requires the use of another imaging system comprising multiple lenses and free space between them (Figure 1(e)). Considering that the key merit of nonlocal metasurfaces for edge detection is the removal of traditional 4*f* systems [26], the necessity of an additional set of lenses is contradictory and undermines the advantage of metasurfaces, the compactness. However, a nonlocal metasurface that provides edge detection in the far-field has yet been reported. Here, we propose a nonlocal metasurface for the far-field edge detection by combining an edge detector and space expander, the reverse concept of recently proposed space compressors or spaceplates (Figure 1(d)) [36], [37], [38], [39], [40], [41]. The metasurface consists of an array of dielectric cylinders on a uniaxial slab with a negative refractive index. The ATF of the cylinder array has a quadratic amplitude profile for *p* polarization and blocks the transmission of *s* polarization and thus accentuates the edge features under unpolarized illumination. Meanwhile, the slab supports a quadratically increasing phase profile, which corresponds to the ATF phase of the free space with a negative propagation length that may reach several orders of magnitude thicker than the slab thickness. This unique optical property allows the edge-enhanced image to be delivered to the camera in the far-field without requiring additional optical setups such as infinity-corrected systems (Figure 1(f)). A lens may be added in front of the metasurface when required to alleviate these issues. We numerically demonstrate that the edge-enhanced image generated by our metasurface has a clear image with significantly less distortion in the far-field, whereas the edge-enhanced image of the conventional edge detection metasurface suffers from severe distortion during propagation. This edge detection in the far-field assisted by nonlocal metasurfaces will relieve the burden of placing optical components closely to the edge-detected images and compactify the analog image processing.

## 2 Result and discussion

### 2.1 Space expander

Our metasurface for the far-field edge detection consists of two parts: an edge detector and a space expander. The main role of each part is to feature edges of a given image and to cancel out the free space propagation so that the edge-detected image can be delivered to the far-field without distortion, respectively. We discuss the space expander first in this section and introduce the edge detector in the preceding section.

**Figure 2:**
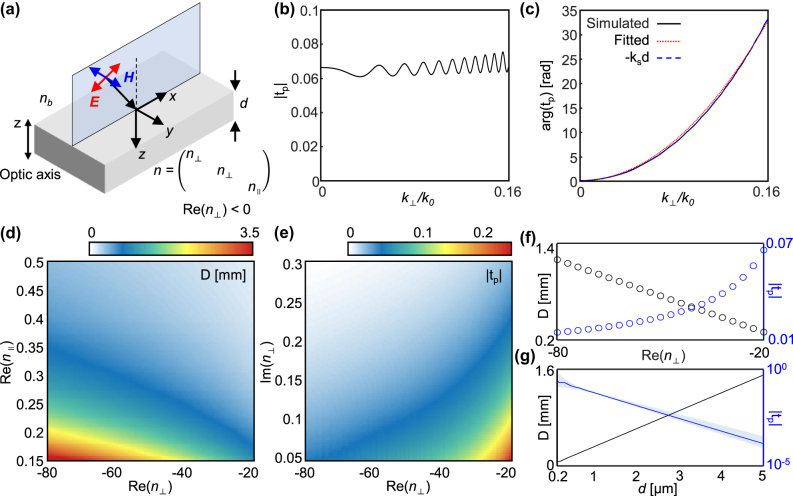
Space expander to deliver the edge-detected image to the far-field. (a) Uniaxial slab that has a negative refractive index along directions perpendicular to the wave propagation as a space expander. (b) Transmission amplitude and (c) phase under *p*-polarized incidence when *n*_*b*_ = 1.45, *n*_⊥_ = −20 + 0.2*i*, *n*_‖_ = 0.269, *λ* = 756 nm, and *d* = 1 µm. (d) The expanded length *D* for various Re(*n*_⊥_) and Re(*n*_‖_) when Im(*n*_⊥_) = 0.2. (e) Averaged transmission amplitude |*t*_*p*_| at 0 ≤ *k*_⊥_/*k*_0_ ≤ 0.6*n*_‖_ for various Re(*n*_⊥_) and Im(*n*_⊥_) when *n*_‖_ = 0.269. (f) *D* and averaged |*t*_*p*_| for various Re(*n*_⊥_) when Im(*n*_⊥_) = 0.2 and *n*_‖_ = 0.269. (g) *D* and |*t*_*p*_| for various *d* when *n*_⊥_ = −20 + 0.2*i* and *n*_‖_ = 0.269. |*t*_*p*_| is plotted on a logarithmic scale, and the range between its maximum and minimum values is shaded.

A uniaxial slab, refractive index tensor of which is expressed as diag(*n*_⊥_, *n*_⊥_, *n*_‖_) can operate as a two-dimensional space expander under *p*-polarized incidence if Re(*n*_⊥_) < 0 and Im(*n*_⊥_) > 0. Here, *n*_⊥_ = *n*_*x*_ = *n*_*y*_ (*n*_‖_ = *n*_*z*_) is the refractive index along the direction perpendicular (parallel) to the optic axis oriented along the *z*-axis (Figure 2(a)). The incident plane is the *zx* plane. We examine the ATF of a slab that has a finite thickness *d* and is embedded in a background medium with the index *n*_*b*_. We first consider the transmission of the slab with the following parameters: *n*_*b*_ = 1.45, *n*_⊥_ = −20 + 0.2*i*, *n*_‖_ = 0.269, wavelength *λ* = 756 nm, and *d* = 1 µm. The results are obtained analytically by solving Maxwell equations at the boundaries and are confirmed by a full-wave simulation conducted using commercial software, COMSOL Multiphysics. The ATF of the slab has weak oscillating amplitude that originates from the thickness comparable to the wavelength (Figure 2(b)) and a quadratic phase profile with positive gradient (Figure 2(c)). It is opposed to the negative phase gradient of the general isotropic medium with a positive index. More specifically, we examine the optical path length of the slab qualitatively by fitting the phase with a curve −*k*_‖_ *D* + *ϕ*_*c*_ (Figure 2(c)). Here, $k_{\|}=\sqrt{k_{0}^{2}-k_{⊥}^{2}}$ is the wave vector along the direction of the optic axis, *k*_⊥_ is the in-plane wave vector, *k*_0_ is the wave number in free space, and *ϕ*_*c*_ is the phase at *k*_‖_ = 0. The fitting parameter *D* is 300 µm. Given that the ATF of the free space for a length of *L* is $\exp\left(ik_{\|}L\right)$ , the phase of which decreases as *k*_⊥_ increases, the increasing phase profile shown in Figure 2(c) indicates that the optical path length of the slab is −*D*. More explicitly, the slab operates as a free space with a negative propagation length (−*D*) and therefore cancels out a free space with the distance *D*. As a result, the slab provides an output image, which would be equal to the input after propagating the free space by length *D*. This corresponds to the inverse operation of the space compression and can be referred to as space expansion. For this reason, we call this fitting parameter *D* an expanded length hereafter. This space expansion is based on a principle similar to that of space compression, in which optical nonlocality is used to replace a thick free-space region with a thin device by mimicking its transfer function, but here, the thickness of the replaced free space is negative.

The space expansion is also expected for an isotropic medium with a negative refractive index, for instance, for an isotropic material with an index of *n*_⊥_. However, *D* in our uniaxial slab is a few orders of magnitude larger than that of the isotropic medium for the following reason. In a uniaxial medium, the wave vector in the slab along the propagation direction follows

(1) $$k_{s}=k_{0}\sqrt{\varepsilon_{⊥}\left(1-\frac{\beta^{2}}{\varepsilon_{\|}}\right)},$$

where $\varepsilon_{b}=n_{b}^{2}$ is the relative permittivity of the background, $\varepsilon_{⊥}=n_{⊥}^{2}$ and $\varepsilon_{\|}=n_{\|}^{2}$ are relative permittivities perpendicular and parallel to the optic axis, respectively, and *β* = *k*_⊥_/*k*_0_ is the propagation constant. The transmission coefficient of the slab can be obtained as

(2) $$t_{p}=\frac{t_{1}t_{2}\exp\left(ik_{s}d\right)}{1+r_{1}r_{2}\exp\left(2ik_{s}d\right)},$$

where *t*_1_ and *r*_1_ are transmission and reflection coefficients from the superstrate to the slab, respectively, and *t*_2_ and *r*_2_ are transmission and reflection coefficients from the slab to the substrate, respectively. Eq. (1) indicates that below the cutoff frequency *k*_cutoff_ = *k*_0_|*n*_‖_|, *k*_*s*_ has a negative real and a positive imaginary part, which induces a negative phase velocity and amplitude attenuation, respectively. As such, $r_{1}r_{2}\exp\left(2ik_{s}d\right)\ll 1$ and thus the phase of *t*_*p*_ is determined dominantly by that of the numerator (Eq. (2)) and becomes approximately *k*_*s*_ *d* (see Figure 2(c), blue dashed curve). This rules out the nonlocality induced by transmission and reflection at the interfaces, leaving the nonlocality due to the propagation as the dominant effect. Such behavior, however, is not always found in media with an isotropic negative refractive index. Since we choose a large |*ɛ*_⊥_/*ɛ*_‖_|, *k*_*s*_ varies rapidly in response to *β* ^2^ than the wave vector in an isotropic medium ( $k_{\|}=k_{0}\sqrt{\varepsilon_{r}-\beta^{2}}$ ) does. This explains why light propagating on the uniaxial slab experiences a negative propagation length, whose absolute value is much higher than *d* or even |*n*_⊥_|*d*.

This phase profile with negative propagation length is also achievable if the medium has a positive index with gain in the *xy* plane, that is, Re(*n*_⊥_) > 0 and Im(*n*_⊥_) < 0. Now *k*_*s*_ in this gain slab is equal and opposite to *k*_*s*_ in the lossy, negative index slab. The negative imaginary part of *k*_*s*_ results in an amplitude of $\exp\left(2ik_{s}d\right)$ larger than unity. Note that *r*_1_ and *r*_2_ are not small enough to cancel out this amplitude due to the impedance mismatch between the slab and the background medium. Therefore, the denominator of *t*_*p*_ can be approximated to $r_{1}r_{2}\exp\left(2ik_{s}d\right)$ and consequently the phase of *t*_*p*_ is approximately −*k*_*s*_ *d* (Eq. (2)), which produces the same effect as before. In contrast, conventional media whose real and imaginary parts of *n*_⊥_ are both positive support positive real and imaginary parts of *k*_*s*_, which makes the denominator of *t*_*p*_ (Eq. (2)) have a negligible phase. Thus, the total phase of *t*_*p*_ is approximately *k*_*s*_ *d*, which has a negative gradient profile. This reasoning involves assumptions, but it perfectly explains the origin of the opposite sign of the phase gradient.

To investigate the effect of refractive indices on space expansion, we examine *D* and the transmission amplitude |*t*_*p*_| averaged at 0 ≤ *k*_⊥_ ≤ *Rk*_cutoff_, where *R* is chosen as 0.6, by varying *n*_⊥_ and *n*_‖_ (Figure 2(d) and (e)). Note that in all our work, the curve fitting is performed at 0 ≤ *k*_⊥_ ≤ *Rk*_cutoff_ because while the phase of *t*_*p*_ has a positive gradient below the cutoff, its shape deviates from the quadratic characteristic at high *k*_⊥_. Figure 2(d) demonstrates that a wide range of *D* can be reached, from a few micrometers to millimeters, by adjusting the real parts of the indices. Considering that the thickness of the slab is only a micrometer, the space expansion effect can be on several orders of magnitude. In particular, *D* increases as both Re(*n*_⊥_) and Re(*n*_‖_) decrease (Figure 2(d)). This agrees well with our previous observation that the gradient of *k*_*s*_ with respect to *β* ^2^ approximately determines *D*. Indeed, Eq. (1) implies that the magnitude of the gradient can be magnified by increasing the absolute value of *n*_⊥_ and by reducing *n*_‖_. This can be explained qualitatively; from the curve-fitting equation, one can deduce that *D* is around

(3) $$|n_{⊥}|d\frac{1-\sqrt{1-R^{2}}}{1-\sqrt{1-n_{\|}^{2}R^{2}}}$$

which shows a nice agreement with the calculated results. The corresponding results are provided in Supplementary Material Section S1. Figure 2(d) only shows for 0.15 < *n*_‖_ < 0.5 for better visualization, but *D* keeps increasing as *n*_‖_ approaches zero. While reducing *n*_‖_ to an infinitesimal value significantly enhances *D* even to a meter scale (for example, *D* = 1 m when *n*_⊥_ = −20 + 0.2*i* and *n*_‖_ = 4.6 × 10^−3^), it simultaneously reduces *k*_cutoff_ and limits this space expansion capability in a small angular regime.

Imaginary part, on the other hand, has less impact on *D*, but mainly affects |*t*_*p*_| (Figure 2(e)). It is consistent with our knowledge that a large imaginary part induces optical losses and thus reduces the total transmission. In addition, as the absolute value of Re(*n*_⊥_) increases, |*t*_*p*_| decreases because the impedance mismatch becomes severe. For better understanding, *D* and |*t*_*p*_| for various Re(*n*_⊥_) when Im(*n*_⊥_) = 0.2 and *n*_‖_ = 0.269 are shown in Figure 2(f). As Re(*n*_⊥_) decreases, *D* increases linearly but |*t*_*p*_| also decreases, degrading the entire efficiency. Lastly, *D* and |*t*_*p*_| for various *d* are shown in Figure 2(g). As *d* increases, *D* increases linearly following the fitting equation *D* = 300.1*d* − 0.2 µm. This linear relation agrees well with Eq. (3) and indicates that a thicker free space can be canceled out by increasing the slab thickness at the expense of reduced transmittance.

While only a one-dimensional profile of ATF is shown in Figure 2(b) and (c), the slab is a rotational-symmetric ATF profile for its uniaxial nature (see Supplementary Material Section S2 for the two-dimensional ATF). This in-plane isotropy results in a two-dimensional, isotropic space expansion of the input image after propagation through *D*. Furthermore, since the sign of *n*_‖_ has no effect on *t*_*p*_ with the zero imaginary part (Im(*n*_‖_) = 0), the space expansion is still achieved when Re(*n*_‖_) < 0, showing a symmetric feature. This indicates that the only requirement of the space expander is to make the sign of the real and imaginary parts of *n*_⊥_ opposite, allowing a wide range of indices along the direction of the optical axis. Previously reported metamaterials that exhibit a negative refractive index or support negative refraction may satisfy this requirement and thus operate as a space expander. For example, the fishnet metamaterial, a well-known negative refractive index metamaterial [42], shows the ATF phase with a positive gradient in the negative refraction regime (see Supplementary Material Section S3). This realistic negative index metamaterial can provide an alternative that replaces the uniaxial slab and enables space expansion. While the value of *D* of the metamaterial is on the order of its thickness and wavelength, this is due to a lack of optimization for space expansion, not because of an intrinsic limitation; *D* can be further increased by tailoring its optical response. In addition, note that our space expander is studied only for *p*-polarized incidence because it will be combined with an edge detector working for *p* polarization while suppressing the *s* polarized components in the following section. This uniaxial slab for the space expander is applicable not only to edge detection but can be combined with other optical elements or systems to cancel out the diffraction during free space propagation.

### 2.2 Combination of the space expander and edge detector

**Figure 3:**
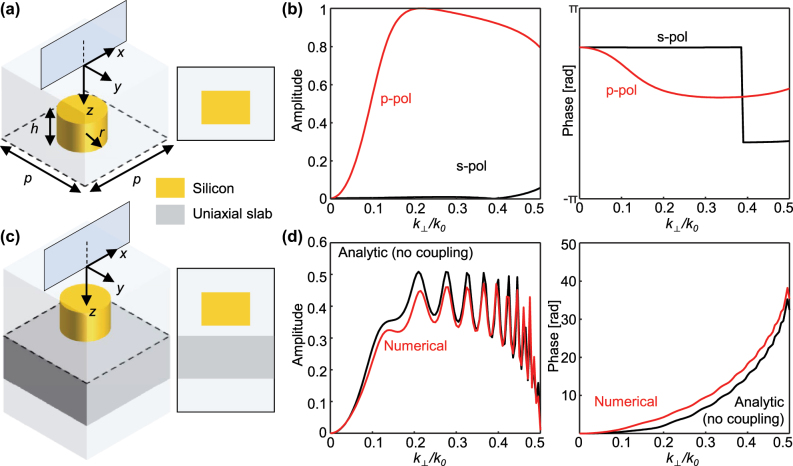
Metasurface design. (a) A metasurface designed for the near-field edge detection and (b) its amplitude and phase. The geometric parameters are given as: periodicity *p* = 376 nm, height *h* = 280 nm, radius *r* = 96 nm. (c) Our metasurface for the far-field edge detection and (d) its amplitude and phase under *p* polarization obtained analytically by assuming no coupling (black) and numerically (red). The phase is set to zero at **k** = 0 for better comparison.

The requirements for the edge detection and space expansions are quadratic amplitude and phase profiles, respectively, which can be satisfied independently without affecting each other. Thus, by combining our space expander with previously reported edge detector designs, the far-field edge detection can be realized. We use the metasurface design reported in ref. [11] as the edge detector after minor modifications (Figure 3(a)). The geometric parameters are given as: periodicity *p* = 376 nm, height *h* = 280 nm, radius *r* = 96 nm. The unit cell is a silicon cylinder embedded in the background medium in a square lattice. The refractive index of the silicon and the background medium are given as 3.74 and 1.45, respectively. The transmission is calculated semi-analytically using a lab-built rigorous coupled-wave analysis code and is also confirmed by the full-wave simulation. This metasurface has a quadratic amplitude profile at low **k**, approximately |*k*_⊥_/*k*_0_| < 0.2, under *p*-polarized incidence and has a negligible transmission under *s*-polarized incidence, i.e., $|t_{p}|\propto {\left(k_{⊥}/k_{0}\right)}^{2}$ and |*t*_*s*_| = 0 (Figure 3(b)). As a result, this edge detector is expected to offer two-dimensional edge detection under unpolarized light. The two-dimensional ATF of this metasurface shows a four-fold rotational symmetry and can be found in Supplementary Material Section S2.

To realize the far-field edge detection, the uniaxial slab that operates as a space expander is inserted below the cylinder array (Figure 3(c)). Transmission of this composite structure under *p*-polarized incidence is obtained using full-wave simulation. To alleviate the high computational cost originating from the large absolute value of the indices, different refractive indices are used: *n*_⊥_ = −5 + 0.2*i*, *n*_‖_ = 0.5. Other parameters are given as the same. The ATF has the amplitude that vanishes at **k** → 0 and oscillates at higher **k** until it reaches the cutoff (Figure 3(d), red). Below this cutoff frequency, the phase has a positive gradient, the unique feature of the uniaxial slab. The curve fitting of this phase profile shows that *D* = 24.76 µm.

The cylinder is separated from the slab by a distance of *λ*/2 to avoid coupling between the slab and the cylinder. If the coupling is indeed absent, the ATF of the whole structure is equal to the pointwise multiplication of the ATF of the cylinder array and that of the uniaxial slab in the **k** space, provided that the diffraction through the spacing is negligible. The analytic results under the coupling-free assumption (Figure 3(d), black) show nice agreement with that of the realistic structure (Figure 3(d), red). In addition, *D* obtained from the analytic ATF is 17 µm.

### 2.3 Comparison between conventional edge detection and our approach

**Figure 4:**
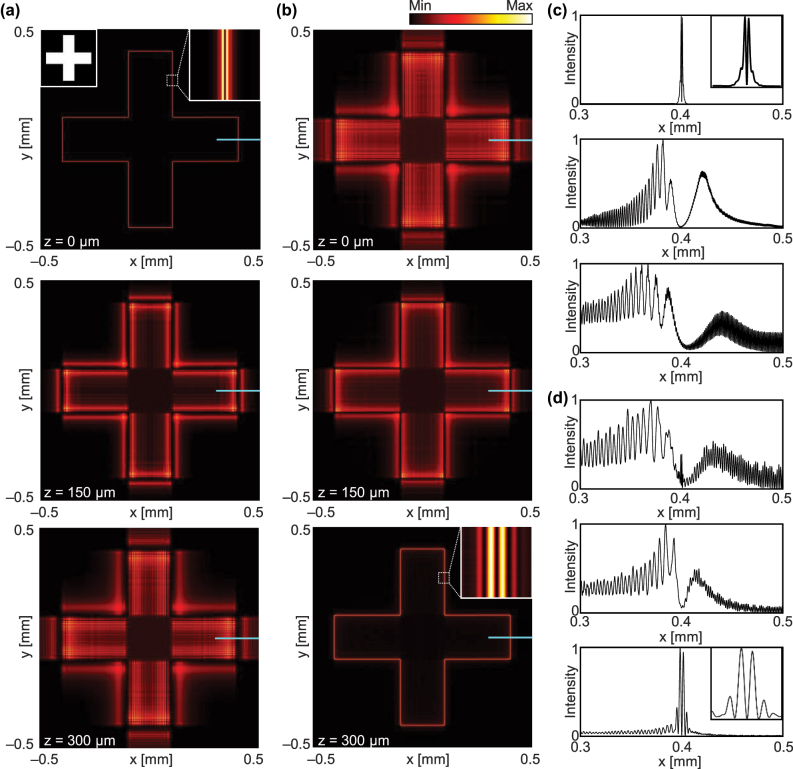
Edge-detected images during propagation. Intensity of beams transmitted through (a) the edge detection metasurface (Figure 3(a)) and (b) our metasurface (Figure 3(c)) at *z* = 0 µm (top), *z* = 150 µm (middle), *z* = 300 µm (bottom). Inset in (a) shows the input image. One-dimensional intensity profiles of the edge-detected images using (c) edge detection metasurface and (d) our metasurface along the cyan lines at *y* = 0. The insets in (c) and (d) show magnified views of the central 10 µm region.

To demonstrate the advantages of far-field edge detection, the evolution of edge-enhanced images generated by our metasurface during propagation is examined and compared with those generated by the conventional edge detection metasurface (Figure 4). For this calculation, we consider unpolarized incidence containing both *p* and *s* polarizations. All parameters are given as in Figure 2(b) and (c), and the corresponding *D* is 300 µm. Note that the analytic two-dimensional ATF is used for the computation (see Figure 3(d) for the validity of this analytic approach). The ATF of this combined structure shows four-fold rotational symmetry with quadratically increasing amplitude and phase profiles until the cutoff frequency for the *p* polarization and negligible amplitude for the *s* polarization (Supplementary Material Section S2).

The conventional metasurface for edge detection provides a clear, edge-enhanced image immediately after the metasurface, i.e., when the propagation distance (*z*) is zero (Figure 4(a), top). The edge is composed of two lines (see inset of Figure 4(a), top), which corresponds to the second-order spatial differentiation of the original input, because of the quadratic amplitude profile (Figure 3(b)). However, the distance between these two line components increases during the propagation (Figure 4(a), middle and bottom), and the overall edge detection quality is degraded in the far-field.

In contrast, the metasurface consisting of the space expander and edge detector that is designed to deliver the edge-enhanced image to the far-field around *z* = 300 µm exhibits blurred images at *z* = 0 µm and *z* = 150 µm (Figure 4(b), top and middle) but provides clear edge features at the target distance (Figure 4(b), bottom). For better visualization, the cross-sectional profiles at *y* = 0 (Figure 4(a) and (b), cyan lines) at each image are shown in Figure 4(c) and (d). Slight degradation of the edge-detected image at *z* = 300 µm (Figure 4(b), bottom) is observed in comparison to the edge-detected image using conventional edge detection metasurface before propagation (Figure 4(a), top) because of the non-constant amplitude, the discrepancy between the ATF phase and its fitting curve, and the nonquadratic phase profile above the cutoff frequency (see Supplementary Material Section S6 for details). Nonetheless, these results confirm that the metasurface consisting of the space expander and edge detector successfully features the edge components and delivers them to the far-field with significantly less distortion. The imaging results at different propagation distances and different slab parameters can be found in Supplementary Material Sections S4 and S5, respectively. Finally, because both the space expander and the edge detector operate in two dimensions, far-field edge detection is enabled in all directions in the *xy* plane.

## 3 Conclusions

In conclusion, we have proposed a nonlocal metasurface composed of an edge detector and a space expander to project the edge-enhanced image to the far-field that reaches several orders of wavelengths. We discuss the principle of the uniaxial slab that operates as the space expander and demonstrate that the slab provides the space expansion effect by adjusting the refractive indices. In contrast to the rapid distortion of the edge-enhanced images generated by conventional edge detection metasurfaces during propagation, our metasurface for the far-field edge detection suppresses the image distortion to the target distance. The optical edge detection in the far-field realized by our metasurface abolishes the use of multiple lenses to deliver the edge-enhanced images to a detector and realizes a true compact, lensless analog image processing.

The far-field edge detection shown in our work is limited to unpolarized incidence because of the polarization dependent properties of the space expander and edge detector. The design of a metasurface that supports the edge detection and space expansion for both *p* and *s* polarizations will enable the edge detection under arbitrary polarization and broaden the applicability. The imaging setup with our metasurface does not require these additional lenses, but this may limit the magnification at the detector. Another limitation that should be solved in future studies is transmission efficiency. While the current metasurface exhibits relatively low transmittance due to the combined effects of edge detection and space expansion, searching for more efficient approaches would be promising. Potential future work will focus on enhancing transmittance and broadening the functionality, including polarization-insensitive operation.

## Supplementary Material

Supplementary Material Details
