# Supplementary material for: A nonlocal metasurface for optical edge detection in the far-field
Source: Nanophotonics. 2025 Sep 19;14(27):5153–61. doi: 10.1515/nanoph-2025-0373 (PMC12717890; doi:10.1515/nanoph-2025-0373)
Supplement: Supplementary file 1 — Supplementary Material Details [file j_nanoph-2025-0373_suppl_001.pdf]

Supplementary Material of
"A nonlocal metasurface for optical edge detection in the far-field"

Doyoon Lee[1], Huu Lam Phan[1, 2, 3], and Minkyung Kim[1*]

[1]Department of Mechanical and Robotics Engineering, Gwangju Institute of Science and Technology (GIST), Gwangju 61005, Republic of Korea

[2]Mechatronics and Robotics Research Group, Faculty of Engineering and Technology, Nguyen Tat Thanh University, Ho Chi Minh 700000, Vietnam

[3]GIST InnoCORE AI-Nano Convergence Institute for Early Detection of Neurodegenerative Diseases, Gwangju Institute of Science and Technology, Cheomdangwagiro 123, 61005 Gwangju, Republic of Korea

[*]m.kim@gist.ac.kr

S1 Theoretical prediction of D

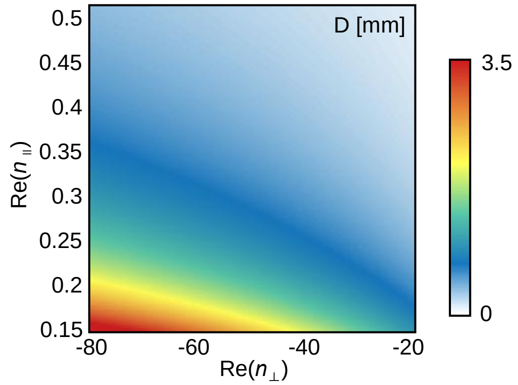

Figure S1. Fitting parameter D obtained from Eq. 3.

The theoretically predicted D (Eq. 3) shows nice agreement with D obtained numerically through the fitting (Fig. 2d).

S2 Amplitude transfer functions

This section shows the two-dimensional ATF used in main text. Fig. S2a and b show the ATF of the edge detector (Fig. 3b) and of the space expander (Fig. 2b and c), respectively. Fig. S2c presents the ATF of our metasurface obtained by multiplying them.

The ATF of the edge detector in Fig. S2a shows a quadratic amplitude profile under p polarization. The ATF of the space expander in Fig. S2b exhibits an oscillatory amplitude profile for p polarization up to the

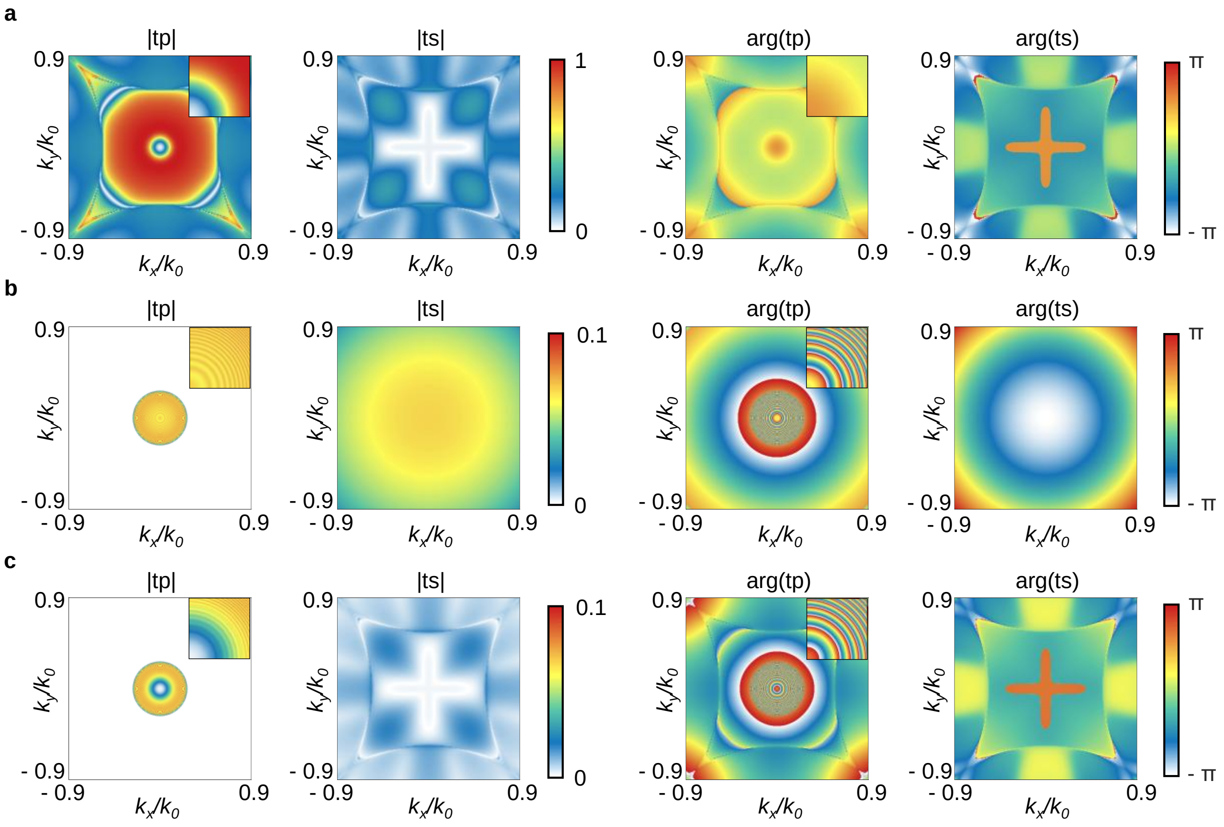

Figure S2. The two-dimensional amplitude and phase profiles of (a) edge detection metasurface, (b) space expander (uniaxial slab), and (c) our metasurface for far-field edge detection. The inset windows show magnified views over $0 \leq k_\perp/k_0 \leq 0.15$.

cutoff frequency ($|k_{\text{cutoff}}/k_0| \leq n_\parallel$), beyond which the amplitude sharply drops to zero. As a result, both amplitude and phase of the combined structure have quadratic profiles under p polarization, enabling the metasurface to simultaneously support the functionalities of both edge detection and space expansion.

S3 A realistic structure with a positive phase gradient

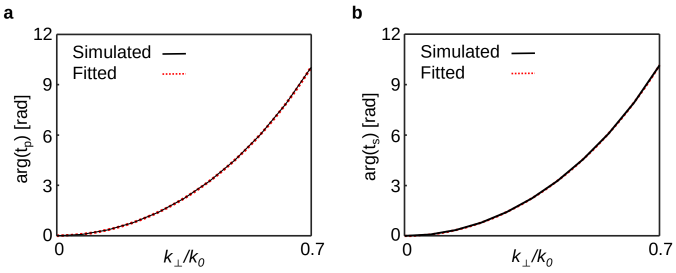

Figure S3. The ATF of a fishnet structure where the target wavelength is 674 nm. One-dimensional phase profiles under (a) p and (b) s polarizations.

To verify the main text's claim that a negative-index metamaterial may have a positive phase gradient, we use the fishnet structure in ref. [1]. The refractive index of Ag is $0.076 + 4.79i$ and that of MgF_2 is 1.38. The phase profiles have a quadratic curve within the range $|k_\perp/k_0| \leq 0.7$, with the fitted D value of 3.77 µm for p polarization and of 3.81 µm for s polarization.

S4 Edge-enhanced images at different propagation distances

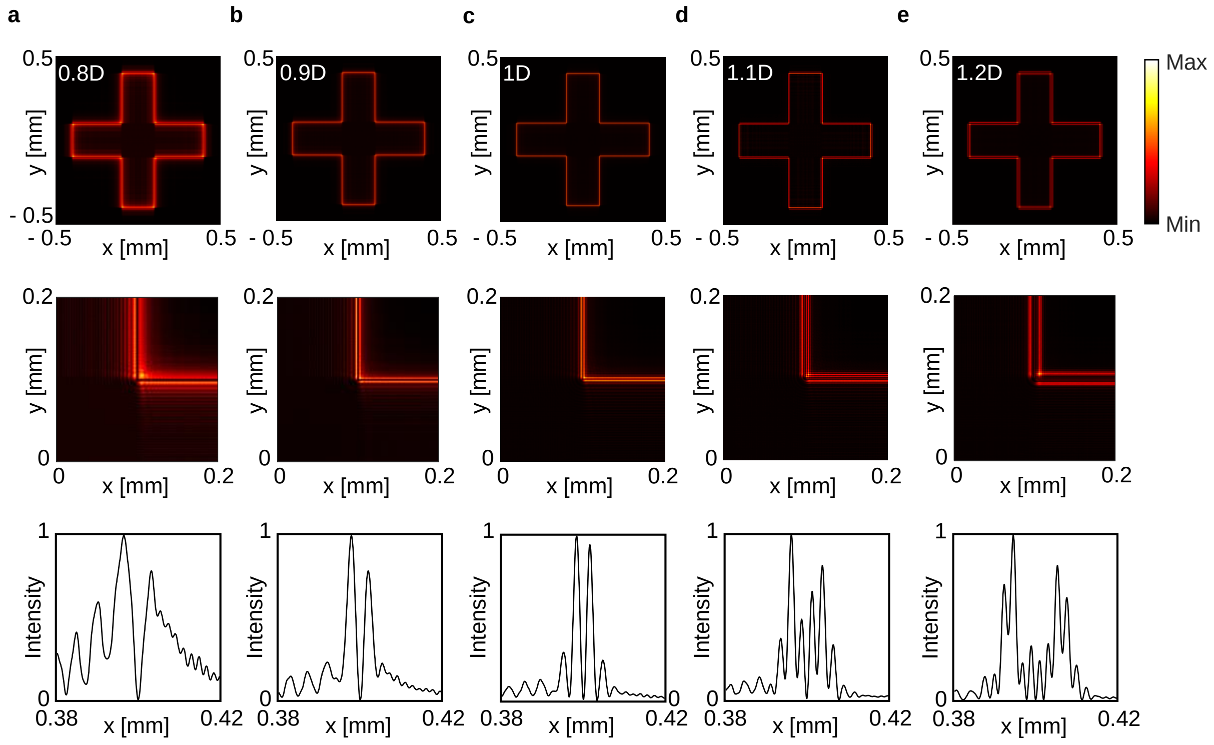

Figure S4. Imaging results for z sweep from $0.8D$ to $1.2D$ by our metasurface. The middle and bottom rows show magnified views and one-dimensional intensity profiles, respectively.

To examine the quality of the edge-enhanced images at different propagation distances, simulations are performed by sweeping z in the range of 0.8–1.2D. The structural parameters and ATF of our metasurface and the value of D are identical to those in Fig. 4. At $z = D$, a clear and sharp edge is detected (Fig. S4c), as demonstrated in the main text. When $z < D$, the edges become blurred (Fig. S4a–b). In contrast, when $z > D$, the peak intensity moves away from the center (Fig. S4d–e). As the distance increases further, this shift becomes more evident, with the peak intensity gradually moving outward from the center.

S5 Edge-enhanced images for different slab parameters

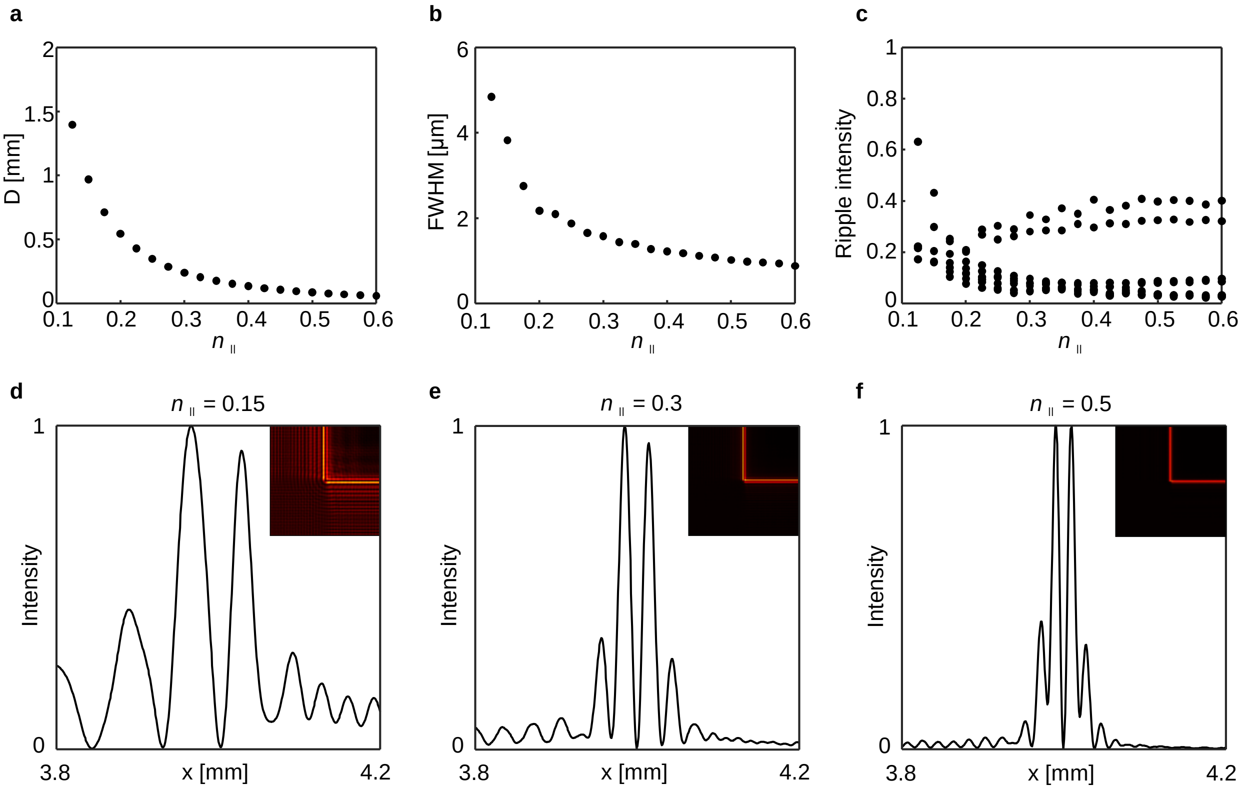

Figure S5. Edge-enhanced images obtained by sweeping $n_{||}$ in the range of $0.1 < n_{||} \leq 0.6$. (a) The fitting parameter D, (b) full-width half-maximum, and (c) ripple intensities. One-dimensional intensity profiles when $n_{||}$ is (d) 0.15, (e) 0.3, and (f) 0.5. The insets present magnified views in the region where x and y range from 0 to 0.2 mm.

In this section, we investigate the quality of the edge-enhanced images for different slab parameters. We first focus on $n_{||}$ (Fig. S5). All other simulation parameters are identical to those in Fig. 4. The propagation distance used in the diffraction theory is determined by Eq. 3. The fitting parameter D agrees well with this input (Fig. S5a). To evaluate the edge resolution numerically, the full-width half-maximum (FWHM) of the edge with the highest peak intensity is calculated (Fig. S5b). In addition, ripple intensities, the peak intensity of the eight strongest ripples excluding the two main edges, are plotted (Fig. S5c). As $n_{||}$ increases, the slab cancels out less space, as manifested by reduced D (Fig. S5a), but the edges becomes sharper (Fig. S5b), with less strong ripples except for two adjacent to the main edges (Fig. S5c).

Whereas we examine the edge detection with the same value of $n_{\perp}$ in Fig. S5, now $n_{\perp}$ is varied to keep D constant using Eq. 3. Within the given range of $n_{||}$ with fixed D, an increase in $n_{||}$ corresponds to a decrease in the real part of $n_{\perp}$. As shown in Fig. S6a, the actual value of D remains nearly constant around 295 μm regardless of the value of $n_{||}$. The FWHM and ripple intensities, except for the two strongest, decrease with increasing $n_{||}$ (Fig. S6b and c).

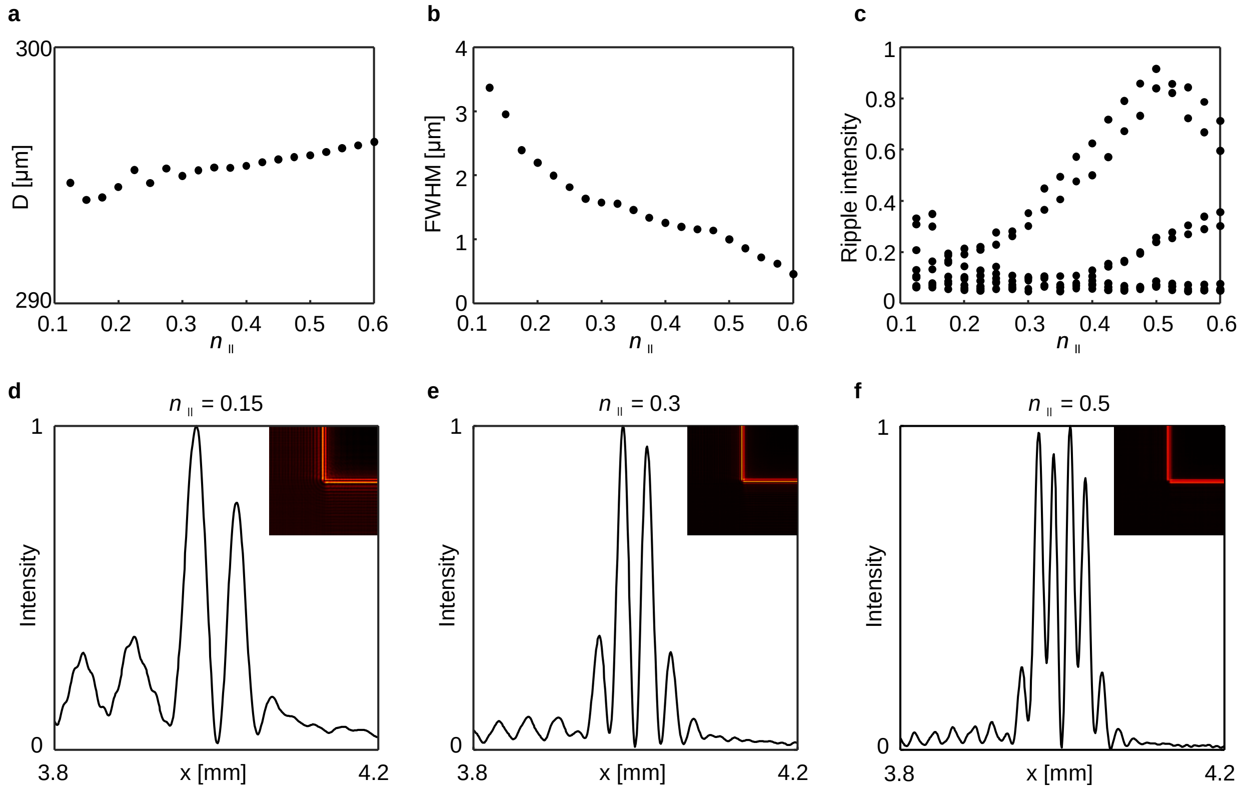

Figure S6. Edge-enhanced images obtained by sweeping $n_{\|}$ in the range of $0.1 < n_{\|} \leq 0.6$ while keeping a constant value of D. (a) The fitting parameter D, (b) full-width half-maximum, and (c) ripple intensities. One-dimensional intensity profiles when $n_{\|}$ is (d) 0.15, (e) 0.3, and (f) 0.5. The insets present magnified views in the region where x and y range from 0 to 0.2 mm.

S6 Edge-enhanced images using different amplitude and phase profiles

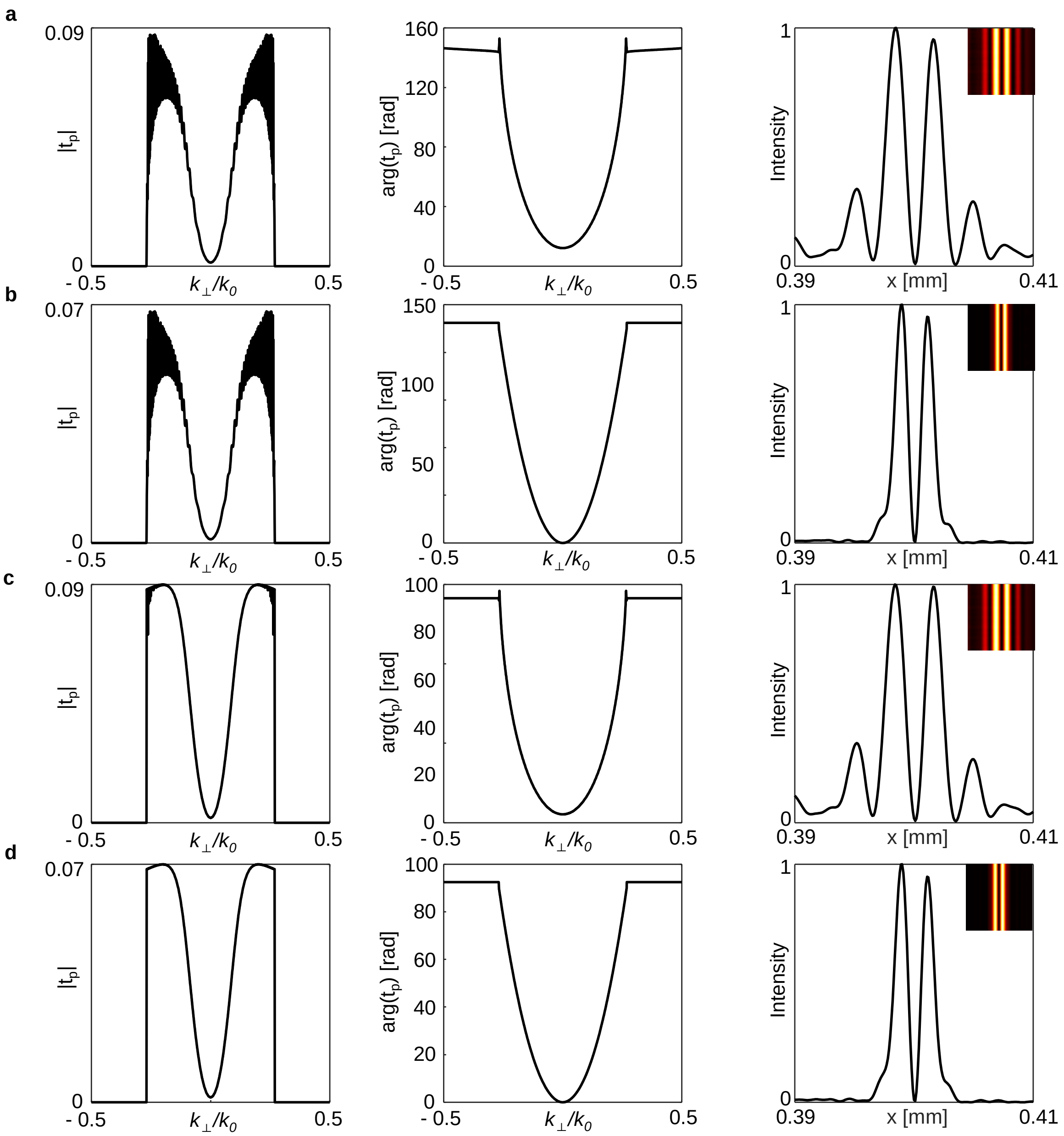

Figure S7. Combined amplitude (left) and phase (middle) profiles and (right) imaging results by using (a) original amplitude and phase profiles, (b) original amplitude and ideal phase profiles, (c) ideal amplitude and original phase profiles, (d) ideal amplitude and phase profiles, and .

Among the three factors of edge quality degradation, the nonquadratic phase profile above the cutoff frequency dominantly affects the edge-enhanced image. To demonstrate this more clearly, we considered the following four cases: (1) original amplitude and phase, (2) original amplitude and ideal phase, (3) ideal amplitude and original phase, and (4) ideal amplitude and phase. Figure S7 shows the transfer functions

and the edge-detected images after free-space propagation by a distance D for the four cases.

We first use the original t_p of the uniaxial slab without any modification (Fig. S7a). We then replace only the phase with its deal counterpart while preserving the original amplitude (Fig. S7b), replace only the amplitude with its ideal counterpart while keeping the original phase (Fig. S7c), and finally replace both amplitude and phase with their ideal values (Fig. S7d). The ideal amplitude is a step function with the slab's average transmission (0.07) up to the fitting cutoff (Rk_{cutoff}) and zero thereafter. The ideal phase has a quadratic profile up to the cutoff. Meanwhile, t_p of the edge detection metasurface (shown in Fig. 3 in our main manuscript) was used without any change. The edge-detected images of four cases are shown in the right column of Fig. S7. All other simulation conditions were identical to those in Fig. 4 except for t_p. Since the space expansion effect of our uniaxial slab appears for p polarization, we only varied t_p during the simulations.

Comparison of these four cases reveals that the dominant cause of the four-line edges is the nonideal phase (Fig. S7c). The original phase with a nonquadratic curve profile at high $\mathbf{k}$ makes waves with different $\mathbf{k}$ components reach with clarity at different propagation planes and generates ripples with non-negligible intensity (Fig. S7a and c). In contrast, with the ideal phase profile, the oscillating amplitude profile or its sudden drop to zero at the cutoff does not degrade the imaging quality noticeably (Fig. S7b).

References

[1] M. I. Aslam and D. Ö. Güney. Dual-band, double-negative, polarization-independent metamaterial for the visible spectrum. *J. Opt. Soc. Am. B* **29**(10), 2839–2847 (2012).
